# Automated Home-Cage Monitoring During Acute Experimental Colitis in Mice

**DOI:** 10.3389/fnins.2021.760606

**Published:** 2021-10-22

**Authors:** Eva Zentrich, Steven R. Talbot, André Bleich, Christine Häger

**Affiliations:** Institute for Laboratory Animal Science, Hannover Medical School, Hannover, Germany

**Keywords:** automated home-cage monitoring, activity, DSS-induced acute colitis, Digital Ventilated Cage, contactless, 24/7

## Abstract

For ethical and legal reasons it is necessary to assess the severity of procedures in animal experimentation. To estimate the degree of pain, suffering, distress or lasting harm, objective methods that provide gradebale parameters need to be tested and validated for various models. In this context, automated home-cage monitoring becomes more important as a contactless, objective, continuous and non-invasive method. The aim of this study was to examine a recently developed large scale automated home-cage monitoring system (Digital Ventilated Cage, DVC^®^) with regard to the applicability and added value for severity assessment in a frequently used acute colitis mouse model. Acute colitis was induced in female C57BL/6J mice by varying doses of DSS (1.5 and 2.5%), matched controls received water only (0%). Besides DVC^®^ activity monitoring and nest scoring, model specific parameters like body weight, clinical colitis score, and intestinal histo-pathology were used. In a second approach, we questioned whether DVC^®^ can be used to detect an influence of different handling methods on the behavior of mice. Therefore, we compared activity patterns of mice that underwent tunnel vs. tail handling for routine animal care procedures. In DSS treated mice, disease specific parameters confirmed induction of a graded colitis. In line with this, DVC^®^ revealed reduced activity in these animals. Furthermore, the system displayed stress-related activity changes due to the restraining procedures necessary in DSS-treatment groups. However, no significant differences between tunnel vs. tail handling procedures were detected. For further analysis of the data, a binary classifier was applied to categorize two severity levels (burdened vs. not burdened) based on activity and body weight. In all DSS-treatment groups data points were allocated to the burdened level, in contrast to a handling group. The fraction of “burdened” animals reflected well the course of colitis development. In conclusion, automated home-cage monitoring by DVC^®^ enabled severity assessment in a DSS-induced colitis model equally well as gold standard clinical parameters. In addition, it revealed changes in activity patterns due to routine handling procedures applied in experimental model work. This indicates that large scale home-cage monitoring can be integrated into routine severity assessment in biomedical research.

## Introduction

In recent years automated home-cage monitoring has become increasingly important in behavioral phenotyping of laboratory mice and might present a tool for the refinement of procedures applied to laboratory animals according to Russell and Burch (1959). The refinement principle of the 3Rs by Russell and Burch (1959) demands to minimize any kind of pain, suffering, distress or harm that animals experience during an experiment. For the recognition of such experiences, good and sensitive parameters are needed. Besides our ethical responsibility to detect any suffering in animals statutory requirements demand researchers to provide classifications of severity (European Union [EU], 2010). However, severity assessment requires methods that include model-specific, objective and gradebale parameters; these have to be tested and validated (Bleich et al., 2020).

To properly record the severity of disease and improve the welfare of the experimental animals, automated home-cage monitoring became an essential element in this process. It enables continuous (24/7) monitoring of the animals (Pernold et al., 2019) that opens the possibility to recognize pain and suffering as quickly and effectively as possible, which is important for robust refinement. This is in line with the recommendations by Hawkins et al. (2011), who provide valuable information on and effective protocols for the welfare assessment. According to the authors, nocturnal animals like many rodents should be observed at night (in their active phase) in order to note relevant changes in behavior. Furthermore, automated home-cage monitoring systems ensure the availability of the recorded data at any time (Pernold et al., 2019). Another big advantage is that the animals remain in their familiar environment (home-cage) eliminating confounding study effects provoked by experimental settings (Richardson, 2015; Bains et al., 2018). In addition, the natural behavior of mice as a prey animal is not affected by the observer and potentially hidden behavior can be detected (Weary et al., 2009).

So far, various methods of automated home-cage monitoring have been developed and established. Besides the use of invasive methods, such as telemetric measurements of vital signs (Cesarovic et al., 2011; Arras et al., 2012), or animal tracking using subcutaneously injected transponders (Lewejohann et al., 2009; Howerton et al., 2012; Weegh et al., 2020), there are already non-invasive automated home-cage methods available. Of particular interest is the recording of mouse behavior and activity without invasive interventions. In previously published studies, technologies such as running wheels (Häger et al., 2018), video recording (Jhuang et al., 2010), microwave-based (Genewsky et al., 2017), or even infrared-based detectors (Dell’Omo et al., 2002) have been used to monitor animal behavior. Recently, an automated home-cage monitoring system (Digital Ventilated Cage, DVC^®^) based on electromagnetic waves has been developed enabling the monitoring of the activity of mice 24/7 in real-time through a capacity-based technology (Iannello, 2019). By using this quite new technology, Pernold et al. (2019) were able to show influences on mice activity due to different time points of routine procedures, such as cage changes. Furthermore, changes in activity patterns in the SOD1G93A mouse model of Amyotrophic Lateral Sclerosis were described (Golini et al., 2020).

Aim of the present study was to evaluate whether the DVC^®^ system enables severity assessment in a mouse model of inflammatory bowel disease (IBD). The major clinical entities of IBD, Ulcerative colitis and Crohn’s disease, are widespread inflammatory diseases of the intestine and represent a significant impairment of the quality of life and performance of those concerned. Besides abdominal pain, diarrhea, and rectal bleeding, also weight loss, fever, and fatigue can occur (Strober et al., 2007). To mimic the disease, various IBD models are available, with the acute dextran sodium sulfate (DSS)-induced colitis mouse model being a frequently used one. DSS results in a disruption of the intestinal barrier accompanied by clinical signs like weight loss and diarrhea (Solomon et al., 2010). The disease severity also depends on the degree of intestinal inflammation (Solomon et al., 2010), and thus it is important to monitor the animals closely. Besides the use of model-specific parameters and the observation of nesting behavior, this study measured the animals’ activity by applying the DVC^®^ system as a large scale automated home-cage monitoring system. We questioned whether changes in activity measured by this system mirror the disease development in the animals and can aid in the categorization of the affected state in this model system.

Additionally, we aimed at comparing activity patterns of mice that underwent tunnel vs. tail handling for routine animal care procedures using the DVC^®^ system. It is already known that mice handled by a tunnel show less anxiety than mice handled by the tail (Gouveia and Hurst, 2013; Nakamura and Suzuki, 2018) and that tunnel handling improves the well-being of animals (Henderson et al., 2020). These studies investigated the behavior of the mice by performing behavioral assays such as the elevated plus-maze or voluntary interaction test (Gouveia and Hurst, 2013; Nakamura and Suzuki, 2018; Henderson et al., 2020). To the authors’ best knowledge it had not been analyzed whether the general activity of mice in their home-cage is also affected by tunnel handling and this has therefore been examined in this study.

## Materials and Methods

### Animals and Housing Conditions

For colitis experiments, thirty female, in-house bred C57BL6/J mice were received from the Central Animal Facility (Hannover Medical School, Hannover, Germany) at the age of 9–10 weeks. Upon arrival the animals were randomly pair-housed and assigned to an experimental group by blindly shuffling and distributing animal score sheets. The animals were given an acclimatization period of 3 weeks to get used to environmental conditions and experimenter by daily handling and weighing. Throughout the acclimatization and experimentation phase the animals were housed in standard individually ventilated cages (IVC; Type GM500, Tecniplast) and had access to autoclaved tap water and standard rodent chow (Altromin 1324 TPF, Altromin Spezialfutter GmbH & Co., KG, Lage, Germany) *ad libitum*. Every cage was provided with the same amount of bedding (120 g of poplar wood, AB 3–10V, Thomsen DeLuxe Bedding “LAB.BED,” Thomsen Räucherspäne Räucherholz GmbH & Co., KG, Handewitt, Germany) and nest material consisting of 10 g folded strips of paper (ANT Pet Bedding, ANT Tierhaltungsbedarf, Buxtehude, Germany) and one cotton roll (ANT cotton rolls, WR 4 12 mm × 38 mm, ANT Tierhaltungsbedarf, Buxtehude, Deutschland). The cages were changed once a week by providing the animals with new bedding and nest material to ensure standardized activity measurement and nest scoring. The mice were monitored and handled under a laminar flow cabinet (Labgard Class II, Laminar Flow, Biological Safety Cabin, NuAire, Inc., Fernbrook Lane, Minnesota, United States) daily to get used to the procedures/experimenter and to closely monitor the state of health during the developing colitis. The daily checks and measurements were always carried out by one and the same female, experienced and trained experimenter, always between 2 and 3 h after the light was switched on. In this process and throughout all other stages of the experiment except for histological evaluation (allocation, experiment, assessment and data analysis) the experimentator was aware of the group allocation. To minimize confounding factors the room was only entered by the one experimenter and the order of the cages for daily examination was arbitrary. In all cohorts, tunnel handling (Plexx, Mouse Tunnel red, #13102, Netherlands) was used for routine care and experimental procedures, except for fresh feces collection (described in more detail in the corresponding section). Routine health surveillance according to Mähler et al. (2014) was conducted using a sentinel system. The mice were housed under a standardized 14/10 h light/dark cycle and the room temperature was maintained at 23 ± 1°C with a relative humidity of 49 ± 9%.

In order to investigate the impact of two different routine handling methods (tail vs. tunnel handling) without further experimental intervention (no DSS induction, Haemoccult^®^ test, nest scoring, or sample collection for histology) additional twenty mice of the same age, strain, and gender were monitored and handled with the assigned method (tail or tunnel). The housing conditions were the same as for the experimental animals.

### Experimental Setup

#### Colitis Model

To induce acute colitis animals were exposed to different dosages of DSS (Lot No.:Q7418, M.W: 36.000–50.000 Da, MP Biomedicals, Illkirch, France) in distilled drinking water for five consecutive days (9 a.m. on day 0 - 9 a.m. on day 5). The set up was as follows: group 1: 0% DSS, control (*n* = 10 animals/5 cages); group 2: 1.5% DSS (*n* = 10 animals/5 cages) and group 3: 2.5% DSS (*n* = 10 animals/5 cages). The control animals continued to receive autoclaved drinking water. At the end of the experiment (d14), all animals were euthanized by CO2 inhalation and subsequent cardiac puncture. Afterward, samples were taken for histological examinations.

#### Histology

At the end of the study, histology of the cecum and colon were prepared. Briefly, the colon was flushed directly with Phosphate-buffered saline (PBS) and placed in a histology cassette as a “Swiss role” (Moolenbeek and Ruitenberg, 1981). Colon and cecum were fixed in a 4% buffered formalin solution. After 4 days the samples were transferred to PBS and the cecum was cut in half and cleared of intestinal contents. After embedding tissue in paraffin blocks, sectioning these blocks, and H&E staining, the samples were scored blinded with regard to inflammation by using a score developed for DSS colitis (Bleich et al., 2004).

#### Clinical Examination

##### Clinical Score

Throughout the experiment, animals were scored daily on the basis of a previously published clinical colitis score (Bleich et al., 2010; Table 1), used for severity assessment of DSS-induced colitis in mice (Häger et al., 2015). The maximum score was set to 20 as described in detail in Table 1. Besides clinical parameters like body weight and fecal consistency also general clinical parameters such as posture or spontaneous behavior were evaluated. The severity of disease was categorized in: score 0 = not, score 1–8 = mildly, score 9–17 = moderately and score 18–20 = severely affected. For statistical analysis, d0 was set as a day of reference, as it represents the condition before DSS treatment.

**TABLE 1:** Clinical colitis score.

Clinical parameters		Score
Weight	0–3% weight loss or weight gain	0
	4–10% weight loss	1
	11–20% weight loss	2
	>20% weight loss	3
Stool consistency	Normal, soft, soft with blood	0–2

**General clinical parameters**		**Score**

Posture	Normal to hunched	0–2
Spontaneous behavior	Normal to no activity (before disturbing)	0–2
Provoked behavior	Normal to no activity (after disturbing)	0–2
Evaluation of the eyes	Clearness, openness	0–3
Evaluation of the fur	Cleanliness, gloss, smoothness	0–3
General appearance	Not, mildly, moderately, severely disturbed	0–3

**Total**		**Score**

	Not	0
	Mildly	1–8
	Moderately	9–17
	Severely affected	18–20

##### Body Weight

Body weight was assessed as a parameter for the clinical scoring as well as for analyzing it separately. A weight loss of more than 18% of the initial body weight was defined as a humane endpoint. Body weights of the last 3 days (day -2 to day 0) of the adaption period were used as bsl and all subsequent data were presented as percentage change from bsl.

##### Fecal Occult Blood Test

In addition to the clinical scoring, the presence of blood in the stool was examined daily (days 0–14) in the three DSS groups. Therefore, a guaiac-based fecal occult blood test (Haemoccult^®^, Beckman Coulter) was performed using fresh feces of the mice. This Haemoccult^®^ test enabled the visualization of occult blood by blue coloration of the test field. In order to receive fresh feces of the animals, the mice were taken out of the cage via tunnel and placed on the cage grid. The base of the tail was then grasped between thumb and forefinger. If the animals did not defecate directly, the anus and abdomen were massaged gently. After feces collection mice were placed back in the cage using the tunnel again.

#### Nest Building Behavior

Throughout the experiment, nest building behavior of the experimental animals was assessed on a non-blinded basis at the time of daily animal checks. Every morning (2–3 h after lights on) nest complexity was scored using the protocol established by Hess et al. (2008). Therefore, the cages were removed from the rack and opened under a lamina flow cabinet. Immediately afterward, the structure of the nests was scored, always by the same observer. In the course of the weekly cage change, every cage was equipped with the same amount of nesting material (120 g of bedding, 10 g of folded strips of paper, and one cotton roll). Bls nest scoring were determined from day -13 to day 0 by calculating the mean of all these days for each cage.

#### Activity Monitoring

In addition to the already mentioned methods that require interaction with the mouse and/or cage and must be carried out in the animal room, the DVC^®^ (Tecniplast, Buguggiate, Italy) was used to monitor the 24/7 activity of the mice. The technology is based on electromagnetic waves. Under each home cage, a sensing board was installed consisting of twelve electrodes to detect differences in the electrical capacitance (every 0.25 s) (Iannello, 2019; Pernold et al., 2019). With the help of a metric so-called Animal Locomotion Index, which was developed and validated by Tecniplast (for further information see Iannello, 2019), interferences of the capacitance are converted into activations and represented in% random unit and normalized (0–100%) (Iannello, 2019). The average activity of each home cage was specified in a 1-min time interval. All data was collected by an assigned computer (DVC^®^ Master) and sent to a web-based software application (Giles et al., 2018; Iannello, 2019). For baseline calculation and data analysis, the metric average of each cage in a 5-min interval was used. Bls activities were determined from day -20 to day 0 by calculating the mean activity (absolute values) of all these days for each cage. For all further analyses, one cage represents one unit.

### Comparison of Handling Procedures

To compare the impact of handling procedures on the animals’ activity, a total of 20 mice were housed in pairs and randomly assigned to two different groups by blindly shuffling and distributing animal score sheets (group 4: tail handling, *n* = 10 animals/5 cages; group 5: tunnel handling, *n* = 10 animals/5 cages). The animals were monitored and handled daily. Just as in the DSS groups, a daily clinical examination including determination of body weight was performed and the activity was monitored 24/7. However, no DSS administration, Haemoccult^®^ test, nest scoring, or sample collection for histology were performed in these animals.

As described in the literature, for tail handling, the experimenter grasped the base of the tail between thumb and forefinger and lifted the mouse gently onto the hand (Hurst and West, 2010; Henderson et al., 2020). In the tunnel handling group mice were guided toward the tunnel. After entering it, the tunnel was lifted up and the experimenters hands were cupped over the ends of the tunnel to avoid escape (Hurst and West, 2010; Henderson et al., 2020).

### Statistical Analysis

All statistical tests were performed using Graphpad Prism 8 software (La Jolla, California). Values are presented as mean ± standard deviation if not stated otherwise. Data were tested against the hypothesis of a normal distribution with the Shapiro-Wilk test. All data, except the histology and clinical colitis score data, approximated a normal distribution. Comparisons between DSS-treated groups and controls were performed with a two-way repeated measure analysis of variance (two-way RM ANOVA) followed by a *post hoc* Tukey test (activity, body weight, nest building) and Kruskal-Wallis test (H) followed by a *post hoc* Dunn’s test (histology and clinical colitis score). Comparisons within groups (from bsl/d0) were performed with a one-way repeated measure analysis of variance (one-way RM ANOVA) followed by a *post hoc* Dunnett’s test (activity, body weight, clinical colitis score). To compare the Haemoccult^®^ test results between the groups, a Fisher’s exact test was performed, and to compare them within each group to d0, the McNemar test was used (R software. V4.0.3, R R Core Team, 2020). During analysis no outlier criteria were identified and no criteria were used for including or excluding animals. For the analysis of histology, clinical colitis score, body weight and fecal occult blood test animals were used as experimental units (*n* = 10 animals for each group). For the analysis of nest building and activity cages were used as experimental unit (*n* = 5 cages each group). *A priori* power analysis was performed. *P* < 0.05 was considered significant with ^∗^: *p* < 0.05, ^∗∗^: *p* < 0.01, and ^∗∗∗^: *p* < 0.001 (varied symbols).

Microsoft^®^ Excel^®^ (v16.0.4549.1000, Microsoft Office Professional Plus 2016, ^©^Microsoft Corporation) was used to create heat maps for activity pattern analysis.

#### Linear Severity Classifier

A model with binary class information based on the variables activity and body weight was trained to classify data into two severity categories. For this, data from day 7 of the colitis model were used as a training set and labeled 1 for the 2.5% DSS group as well as 0 for the rest. The model was trained with a generalized linear model from the binomial family with categorical outcomes and a logit link function. The probabilities on the logit scale were used in class predictions at the *p* > 0.5 threshold. Data from other days of the colitis model were then used as test data to evaluate the classification performance of the classifier. These calculations were performed in the R software (v4.0.3, R Core Team, 2020).

## Results

### Model-Specific Parameter Describing Colitis Manifestation

#### Histology

**FIGURE 1:**
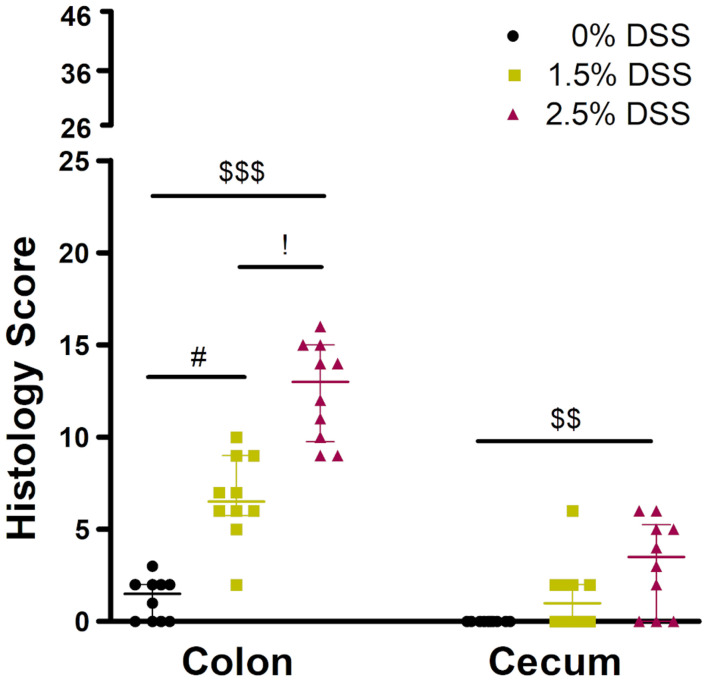
Histology of the three groups of the DSS-induced colitis model. The histology score of the colon ranged from 0 to 46 and of the cecum from 0 to 23. *N* = 10 animals in each group. Comparison between the groups: Kruskal-Wallis Test (colon: *H* = 24.17, *p* < 0.0001; cecum: *H* = 10.93, *p* < 0.0042) + Dunn’s *post hoc* test; #: 0% vs. 1.5% (adj. *p* < 0.0357), $$: 0% vs. 2.5% (adj. *p* < 0.0029), $$$: 0% vs. 2.5% (adj. *p* < 0.0001), !: 1.5% vs. 2.5% (adj. *p* = 0.4754). Values are presented as median with IQR.

For the analysis of a graded acute colitis induction, histologies of the intestines were taken from animals treated with 0% (water only), 1.5 or 2.5% DSS on day 14 at the end of the experiment. Kruskal-Wallis test showed significant differences in the histology score between different DSS concentrations (colon: H = 24.17, *p* < 0.0001; cecum: H = 10.93, *p* < 0.0042). Dunn’s *post hoc* test revealed that colons of DSS-treated mice showed significantly higher scores with a median score of 6.5, IQR = 5.75–9.0 (1.5% DSS group, adj. *p* < 0.0357) and 13.0, IQR = 9.75–15 (2.5% DSS group, adj. *p* < 0.0001) out of a maximum score of 46, when compared to control animals (median score: 1.5, IQR = 0–2) (Figure 1). Analysis of the cecum still revealed a median score of 3.5, IQR = 0–5.2 out of a maximum score of 23 in the 2.5% DSS group, a significantly higher score compared to the 0% DSS group (median score 0, IQR = 0–0, 0% vs. 2.5%: adj. *p* < 0.0029).

#### Clinical Examination

**FIGURE 2:**
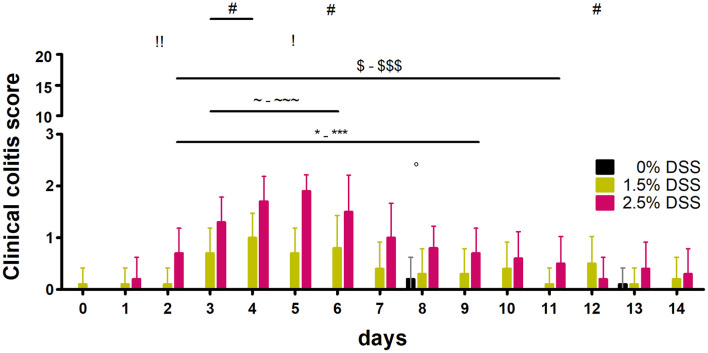
Results of the clinical colitis score. Clinical colitis score of all three groups of the DSS-induced colitis model (*n* = 10 animals in each group). Comparison to d0: one way RM ANOVA + Dunnett’s *post hoc* test;°: 0% [*F*_(__14_, _126__)_ = 1.678, *p* = 0.0682; d8: adj. *p* = 0.0154], ∼-∼∼∼: 1.5% [*F*_(__14_, _126__)_ = 4.094, *p* < 0.0001; d3: adj. *p* = 0.0428, d4: adj. *p* = 0.0004, d5: *p* = 0.0428, d6: adj. *p* = 0.0104], *-***: 2.5% [*F*_(__14_, _126__)_ = 14.08, *p* < 0.0001; d2: adj. *p* = 0.0189, d3-6: adj. *p* < 0.0001, d7: adj. *p* = 0.0002, d8: adj. *p* = 0.0045, d9: *p* = 0.0189]. Comparison between the different groups: Kruskal-Wallis Test + Dunn’s *post hoc* test; #: 0% vs. 1.5% (d3: adj. *p* = 0.0237, d4: adj. *p* = 0.0101 and d6: adj. *p* = 0.0259), $-$$$: 0% vs. 2.5% (d2: *p* = 0.0015, d3-d6: *p* < 0.0001, d7: adj. *p* = 0.0008, d9: *p* = 0.0033, d10: *p* = 0.0154, d11: adj. *p* = 0.018), !, !!: 1.5% vs. 2.5% (d2: *p* = 0.0086, d5: *p* = 0.01).

Using the model-specific clinical colitis score, the parameters body weight, fecal consistency, posture, behavior (spontaneous and provoked), and appearance (fur and eyes) of the animals were recorded and results are shown in Figure 2. Within group comparisons over time revealed no statistically significant changes in the 0% DSS group [one-way RM ANOVA; *F*_(__14_, _126__)_ = 1.678, *p* = 0.0682] but significantly increased scores in 2.5% [one-way RM ANOVA; *F*_(__14_, _126__)_ = 14.08, *p* < 0.0001] and 1.5% DSS-treated mice [one-way RM ANOVA; *F*_(__14_, _126__)_ = 4.094, *p* < 0.0001]. A maximum individual score of 3 out of 20 (day 6) in the 2.5% DSS group and 2 out of 20 in the 1.5% DSS group (day 4 and 6) was detected (Figure 2). Dunnett’s *post hoc* test revealed significant increases in the DSS-treated mice with the highest mean values on day 5 in the 2.5% DSS group (1.9 ± 0.31, adj. *p* < 0.001) and on day 4 in the 1.5% DSS group (1.0 ± 0.47, adj. *p* < 0.0004) (Figure 2). During the experiment, the 2.5% DSS-treated mice showed more significant differences compared to d0 than the 1.5% DSS-treated mice (2.5% group for 8 days: days 2–9; 1.5% group for 4 days: days 3–6) (Figure 2). Statistically significant differences were identified between all treatment groups from day 2 to day 12 with biggest difference on day 5 (*H* = 24.62, *p* < 0.0001; Dunn’s *post hoc* test: 0% vs. 2.5%: adj. *p* < 0.0001, 1.5% vs. 2.5%: adj. *p* < 0.01). Dunn’s *post hoc* test revealed significant differences between the 0 and 1.5% DSS groups on days 3, 4, and 6 and between the 0 and 2.5% DSS groups from days 2 to 11 (inter-group differences for individual days are depicted in Figure 2). Throughout the experiment, no animal showed changes in posture, behavior, or appearance, but body weights were decreased in the 2.5% DSS group when compared to bsl level [one-way RM ANOVA; *F*_(__14_, _126__)_ = 9.852, *p* < 0.0001] and stool consistency changed in DSS treated animals (1.5 and 2.5% groups). The fecal consistency was the major factor regarding the increased clinical score, as the feces became softer and showed traces of blood with continued DSS treatment. Haemoccult^®^ testing confirmed these findings (Supplementary Figure 1). Individual parameters of the clinical score and body weight curves are shown in Supplementary Figure 1.

### Nest-Building Behavior

In addition to clinical scoring, the nest-building behavior was monitored to determine severity-related differences. Throughout the experiment, neither significant differences between the groups [two-way RM ANOVA; *F*_(__2_, _12__)_ = 0.3601, *p* = 0.7049] nor interactions between groups and time [two-way RM ANOVA; *F*_(__28_, _168__)_ = 1.268, *p* = 0.1808] were detected. Score value data can be seen in Supplementary Table 1.

### Automated Home-Cage Monitoring

#### Activity Patterns and Quantification of General Activity

**FIGURE 3:**
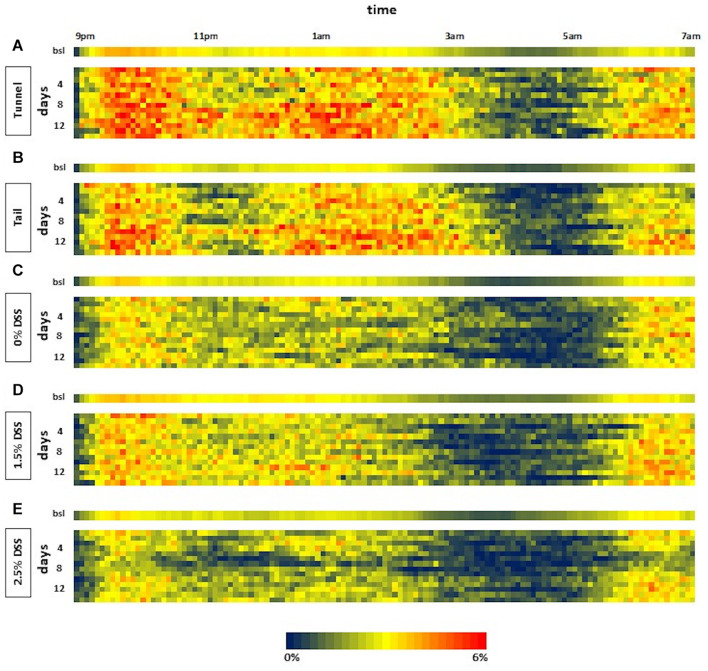
Heat maps of the dark phase from days 1 to 14 of the three DSS groups and the two handling groups. The heat map shows the activity data displayed in 5-min intervals during lights-off for days of bsl and during the experiment (days 1–14). Each line represents one dark phase (9 p.m. to 7 a.m.). The values are color-coded with blue representing low and red representing high activity (0–6%). **(A)** Tunnel; **(B)** Tail; **(C)** 0% DSS; **(D)** 1.5% DSS; **(E)** 2.5% DSS; *n* = 5 cages each group.

**FIGURE 4:**
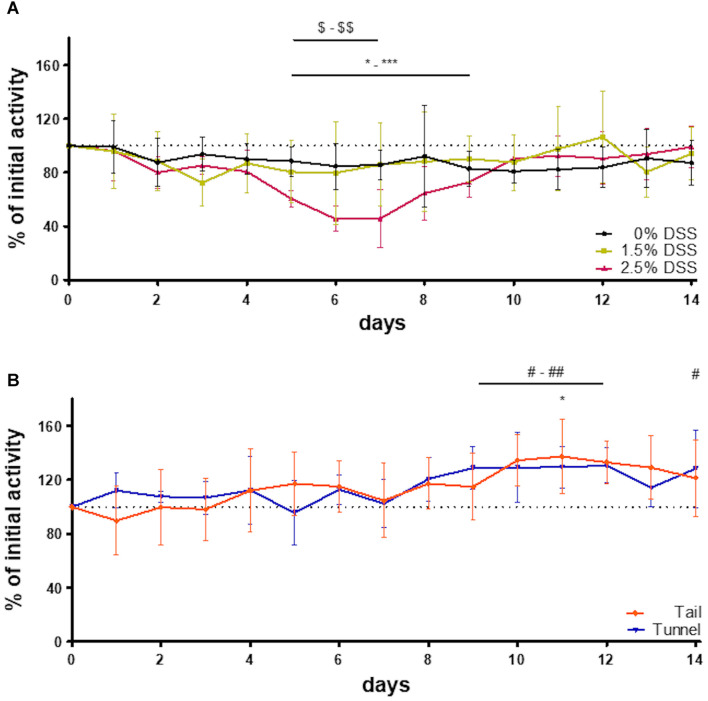
Percentage change of initial activity of the experimental animals and the two handling groups. Percent of initial activity **(A)** of all three groups of the DSS-induced colitis model (*n* = 5 cages each group) and **(B)** of the two handling groups (*n* = 5 cages each group). Compared to bsl: one way RM ANOVA [tail: *F*_(__14_, _56__)_ = 2.700, *p* = 0.0043; tunnel: *F*_(__14_, _56__)_ = 3.703, *p* = 0.0002] + Dunnett’s *post hoc* test; *-***: 2.5% DSS, *: tail, #-## and #: tunnel. Comparison between the groups: DSS groups ($-$$): two-way RM ANOVA [time × group factor: F_(28, 168)_ = 2.354 *p* = 0.0004, group factor: F_(2, 12)_ = 0.7474 *p* = 0.4944]; handling groups: two-way RM ANOVA [time × group factor: F_(14, 112)_ = 1.039, *p* = 0.4212, group factor: F_(1, 8)_ = 0.002775, *p* = 0.9593].

The automated home-cage monitoring system DVC^®^ was used for the analysis of the impact of acute DSS colitis on activity patterns in mice and for the analysis of possible differences in activity due to different handling methods. By applying this system, dark-phase (9 p.m. to 7 a.m.) activity patterns in 5-min intervals were assessed. In heat maps illustrating activity, the blue color represents time intervals of low activity and the red color represents time intervals of high activity. Heat maps of all three DSS groups and the two handling groups, consisting of bsl bars and activity patterns from days 1 to 14, were created (Figures 3A–E). Activity patterns of the DSS groups showed the first peak of activity within the first 2 h after lights were switched off. Approximately 5 h after lights-off a marked decrease in activity was visible. This inactive phase lasted about 2–3 h. Subsequently, another active phase could be detected. It was noticeable that the heat map of the 2.5% DSS group revealed decreased activity (indicated by multiple blue areas) during the whole dark phase on days 5 to 7 (Figure 3E). The statistical analysis confirmed a significant decrease in the 2.5% DSS group from days 5 to 9 compared to bsl [one-way RM ANOVA; *F*_(__14_, _56__)_ = 10.22, *p* < 0.0001; mean difference: 27.3–54.56%; Dunnett’s *post hoc* test: d5: adj. *p* = 0.00015; d6–d7: adj. *p* < 0.0001; d8: adj. *p* = 0.0006; d9: adj. *p* = 0.014] (Figure 4A). Moreover, two-way repeated measure ANOVA revealed a significant time x group interaction [*F*_(__28_, _168__)_ = 2.354, *p* = 0.0004]. Tukey’s *post hoc* test revealed a decreased activity in the 2.5% DSS group compared to the control group from days 5 to 7 (d5: adj. *p* = 0.0055, d6: adj. *p* = 0.0095, d7: adj. *p* = 0.0246). However, no significant differences between the 0% DSS and 1.5% DSS group were observed throughout the experiment.

To check whether the system is also sensitive enough to display differences between handling methods, activity patterns of mice during routine husbandry handled via tail or tunnel were created (Figures 3A,B). Similar to the experimental animals, heat maps showed the first peak of activity within the first 2 h after lights were switched off. Here, the marked decrease in activity was visible approximately 1 h later than in the DSS groups. The inactive phase lasted about 2 h. Subsequently, another active phase could be detected. When comparing the activity pattern of the tail and tunnel-handled group, there was a slight tendency toward more blue areas (inactive intervals) in the tail group (Figure 3B). However, no significant differences between the two handling methods could be detected by statistical analysis [two-way RM ANOVA; time × group factor: *F*_(__14_, _112__)_ = 1.039, *p* = 0.4212; group factor: *F*_(__1_, _8__)_ = 0.002775, *p* = 0.9593; Figure 4B]. Nevertheless, a slight, non-significant tendency toward higher activity in the tunnel-group was observed by comparing the cumulative activity over the whole observation time [two-way RM ANOVA; time × group factor: *F*_(__14_, _112__)_ = 1.339, *p* = 0.1960; Supplementary Figure 2].

Interestingly, the 0% DSS group showed less activity during the experimental phase when compared to the activity patterns of the two handling groups (heat maps in Figure 3) or compared to bsl level (Figure 4A). However, statistical analysis revealed no significant decrease [one-way RM ANOVA; *F*_(__14_, _56__)_ = 0.8806, *p* = 0.5829]. In comparison, the general activity of the handling groups remained around or above bsl. Statistical analysis revealed no significant decrease during the observation time but a slight increase over the bsl level [one-way RM ANOVA; tail handling group: *F*_(__14_, _56__)_ = 2.700, *p* = 0.0043; tunnel handling group: *F*_(__14_, _56__)_ = 3.70, *p* = 0.0002; Figure 4B].

#### Classification of Severity With a Linear Classifier

**FIGURE 5:**
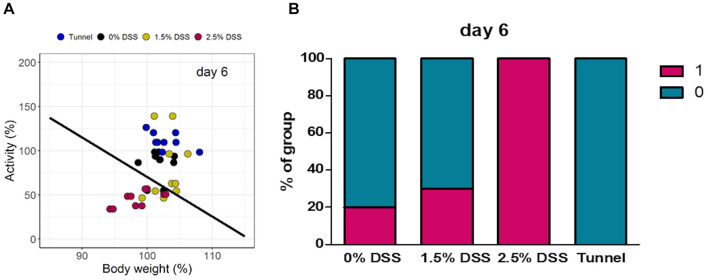
Results of the linear classifier. Data points of all animals based on body weight and activity on day 6. **(A)** Localization of data points of individual mice and **(B)** percentage distribution of each group in classes 0 and 1.

For objective severity classification and prediction, we trained a linear classifier with a generalized linear model from the binomial family (logistic regression). To capture the most extreme severity information of the colitis model, activity and body weight data from day 7 of the experiment were used as training data (Supplementary Figure 3). In this set, data from the 2.5% DSS group were class-labeled 1 (indicating severity) and the remaining data from the other groups were labeled 0 (indicating no or less severity). With the coefficients of the resulting model, a line was drawn into the two-dimensional variable space of activity and body weight that represented the decision boundary of the classifier, trained on day 7 colitis data. The line was then used as a visible discriminator for distinguishing between burdened and unburdened data (classes 0, and 1) (Figure 5A). In subsequent steps, projections of untrained (test) data into the same space allowed predictions of severity classes for each data point. At day 6, differences were clearly visible between various experimental groups in terms of severity classifications. In all three experimental groups, fractions of animals were categorized into severity class 1. The proportion of class 1 was highest in the 2.5% DSS group with 100% (Figure 5B), followed by the 1.5% DSS group with 30%, and the 0% DSS group with 20% (Figure 5B). In comparison to the DSS groups, the tunnel-handled group was classified completely into class 0 (Figure 5B). Categorized data of all days are shown in Supplementary Figure 4.

## Discussion

Animal welfare and the refinement of experimental procedures are fundamental aspects of biomedical research and require an improved and evidence-based severity assessment strategy (Bleich and Tolba, 2017; Bleich et al., 2020). Here, among disease specific parameters like body weight and clinical colitis score, as well as scoring of the nest-building behavior, we assessed the impact of acute DSS-colitis induction on the animal by using an automated home-cage monitoring system (DVC^®^). Expectedly, common clinical scoring revealed an increase in the clinical colitis score in the DSS-treated mice, which correlated with increased DSS doses. Corresponding control animals showed no significant signs of impairment. The automated home-cage monitoring also detected prolonged inactivity phases and significant decreases in activity due to higher concentrations of DSS. Interestingly, a non-significant decrease in general activity in the control group was indicated during the whole experiment when compared to baseline measurements.

According to the literature, clinical scoring, which includes measuring body weight or recording behavioral changes, is a fundamental tool for severity assessment in animal experimentation (Keubler et al., 2018) and requires model-specific adjustment (Morton and Griffiths, 1985). Also in this study model-specific clinical scoring was performed to assess the animals’ welfare during the experiments, which was also used in previous studies (Häger et al., 2015). In the present study, the parameters that contributed most to the increased score values were body weight (in the 2.5% DSS group only) and fecal consistency. This is in line with the recent literature showing reduced body weight (Chassaing et al., 2014; Häger et al., 2015) and changed fecal consistency (Solomon et al., 2010) in mouse models of acute colitis. However, other parameters of the clinical score, such as posture, behavior, and appearance showed no alterations. Since mice are prey species, they may hide natural behavior in the presence of humans (Weary et al., 2009). Furthermore, appropriate training and experience of the observer performing clinical scoring is required. Thus, the clinical score is rather limited in its applicability and is perhaps not sensitive enough to assess subtle changes (Hawkins et al., 2011). Moreover, necessary procedures required for clinical investigation (e.g., restraining for feces collection) are generally disruptive to the animal during its sleep cycle, are potentially stressful, and may only provide a snapshot of the animal’s current condition.

In the present study, nest scoring as a non-invasive method was used additionally to assess the severity of disease. However, in the present study, no significant decrease in nest building activity due to DSS treatment was detected. One explanation could be the relatively mild colitis development does not affect the nest building behavior of the animals. Also the sample size could be a limiting factor for identifying statistically significant differences, though this is very unlikely to be the cause in the present study considering the almost identical nest scores in all treatment groups. Published evidence confirms the suitability of this method for the assessment of distress and pain, e.g., after laparotomy or exposure to isoflurane (Arras et al., 2007; Gjendal et al., 2020). However, not only did our study show no significant effects on nest building behavior due to pain and distress, but also a recent study by Hohlbaum et al. (2017) detected no significant effect of isoflurane exposure on nest building behavior. In this context it is important to consider that the protocols used for nest scoring often vary between the studies.

The focus of the present study was the application of DVC as a non-invasive, contactless and continuous method to investigate changes in activity due to DSS treatment. We observed that mice showed the peak of activity within the first 2 h after lights off, followed by a phase of high activity for an additional 4 h. After this active phase, the mice showed an obvious reduction in activity. This resting time lasted around 2 h, following by a further active phase before lights switched on again. These findings are in line with a study by Pernold et al. (2019) investigating the activity of group-housed female C57BL/6J mice in the DVC^®^ system at three different locations. Interestingly, during the entire period of activity measurement, mice of the DSS treatment groups showed less activity compared to the bsl level than mice of the handling groups. A major difference regarding the handling procedures between the DSS groups and the two groups in routine husbandry was the additional restraining for Haemoccult^®^ testing. Routine procedures in laboratory facilities, like handling and restraint are known as potential sources of stress (Balcombe et al., 2004; Meijer et al., 2006) and can influence the activity of mice (Pernold et al., 2019). In this study, such stress-related differences due to additional restraining were detected by home-cage monitoring. Furthermore, the system revealed more inactive time intervals and an enlarged inactive phase due to DSS induction and a significant reduction in general activity. Other home-cage systems using voluntary wheel running for activity monitoring (Häger et al., 2018; Weegh et al., 2020), revealed a dose-dependent decrease in activity due to DSS administration. However, in the present study, no dose-dependent differences in the animals’ activity were observed, and in comparison the manifestation of the colitis in the present study was milder. Another reason could be the difference between the two methods, measurement of wheel-running activity in the home-cage vs. general activity in the home-cage. In a study (Bains et al., 2018) investigating differences between motivated running-wheel behavior and RFID-based home-cage activity, the authors described the limited detection of wheel-running behavior, since it is part of motivated running behavior. This study suggests that initially, elective activity of the animals decreases before general home-cage activity. Another reason could be the fact that this home-cage system is only able to display the average of the cage activity, while activity of individual mice is unknown. Individual animal tracking can be achieved, e.g., using RFID-based systems (Lewejohann et al., 2009; Weegh et al., 2020), however, these systems usually require an invasive intervention to implant RFID chips. An overview and critical discussion of this technology has recently been given by Voikar and Gaburro (2020).

Moreover, as we observed impacts of handling procedures in the DSS groups, we analyzed tunnel vs. tail handling during routine husbandry to answer the question of whether this DVC^®^ system is sensitive enough to detect subtle differences between groups. In the published literature, mice handled by a tunnel showed less anxiety than mice handled by the tail (Gouveia and Hurst, 2013; Nakamura and Suzuki, 2018) and tunnel handling contributed to improved animal well-being (Henderson et al., 2020). These studies investigated the behavior of the mice by performing dedicated assays such as the elevated plus-maze, open field test or voluntary interaction test (Gouveia and Hurst, 2013; Nakamura and Suzuki, 2018; Henderson et al., 2020). To the authors’ best knowledge it is not yet known whether the general activity of the mice in the home-cage is also less affected by tunnel handling. The results of the DVC^®^ system in the present study revealed no significant differences between mice handled by tunnel compared to mice handled by the tail, however, a slight tendency toward higher activity in the tunnel handled group was observed. These findings revealed that the general activity in the home-cage is not effected by the handling method, even if according to the literature tunnel handling in provoked situations outside the home-cage (e.g., open field test) improves animal well-being (Nakamura and Suzuki, 2018; Henderson et al., 2020). However, it is advisable to prefer tunnel handling to tail handling, as it has already been shown that it contributes to improved interaction with the experimenter and reduces anxiety (Nakamura and Suzuki, 2018).

With the training of a linear classifier, a severity classification of two classes based on both, activity and body weight was possible with the current data. The application of the algorithm demonstrated a graded colitis and mirrored well the course of colitis induction and remission. Furthermore, not only did the DSS-treated groups display fractions in severity class 1 (burdened), this was also observed for the 0% DSS group. In contrast, the tunnel-handled mice in routine husbandry remained entirely at class 0. With the current limited training data, a simple binary model appeared feasible. However, with further data from other studies a more complex, stratified and cross-validated model may lead to a more generalizable characterization of severity levels of colitis by activity data in the near future.

The DVC^®^ reflects several advantages inherent to automated home-cage monitoring systems, which ultimately can contribute to higher reproducibility and better refinement of studies by reducing human bias and interferences (Voikar and Gaburro, 2020). One of the main advantages is the continuous monitoring of the animals (Pernold et al., 2019). Nocturnal animals can be observed in their active phase and any changes in behavior during this time can be detected (Hawkins et al., 2011). In comparison, clinical scoring can only capture a brief moment of the animal’s condition. Furthermore, clinical scoring or behavioral testing are mostly dependent on the presence and/or interaction of the observer. In contrast, the DVC^®^ is a home-cage-based method, thus no further experimental settings are required (Richardson, 2015; Bains et al., 2018). Consequently, the natural behavior of mice as a prey animal is not affected and potentially hidden behavior can be detected (Weary et al., 2009). Moreover, a review by Voikar and Gaburro (2020) gives evidence for the application of the DVC^®^ system for mouse phenotyping. The authors of the present study also see an added value in the system with regard to mouse phenotyping in the colitis models. E.g., behavioral data can aid in the detection of differences in the susceptibility to colitis between mouse strains, as known for the DSS (Mähler et al., 1998) or interleukin-10-deficiency model (Keubler et al., 2015). Recently, a study by Forster et al. (2021) showed limited correlation of the parameter body weight and histological score in the DSS model. Likewise, we have observed that general clinical symptoms do not necessarily correlate with histological scores in colitis models (unpublished). This suggests that it is important to consider also other parameter like activity in order to better capture the severity of disease through multimodal monitoring. This also applies to translational research when it comes to yet ignored aspects of disease in mouse models. It has been known that patients with IBD can develop symptoms such as fatigue syndrome (Nocerino et al., 2020), to our knowledge a component not yet included in phenotyping of models in therapeutic studies. This can possibly be achieved using contactless home-cage monitoring.

However, the system still shows some limitations like the above mentioned fact that it only displays the average of the cage activity. In addition, many parameters for phenotyping, including severity assessment, are model-specific. Therefore, it is advisable to test the applicability of this system in further animal models. Nevertheless, in the authors’ opinion, further developments of such automated systems will enable large-scale, contactless home-cage monitoring in experimental and even non-experimental settings, which represents a considerable added value for animal welfare.

In summary, we have shown in this study that the DVC^®^ system detected significant decreases in activity due to higher concentrations of DSS and changes in activity patterns due to handling procedures. By applying a regression model using the DVC^®^ data, in this experiment a classification into burdened and not burdened animals could be made detectable. This quite new DVC^®^ system of course cannot replace routine clinical examination of the animals, as we have the duty of care for them, but it serves well as an additional refinement method to objectively monitor animal activity and might enable additional stress-inducing procedures like the Haemoccult^®^ test or even weighing to be omitted during the experiments.

## Data Availability Statement

The raw data supporting the conclusions of this article will be made available by the authors, without undue reservation.

## Ethics Statement

This animal study was reviewed and approved by the Lower Saxony State Office for Consumer Protection and Food Safety (LAVES) (License No. 33.19-42502-04-17/2685) and was in accordance with the applicable laws and regulations (German law for animal protection and EU directive 2010/63).

## Author Contributions

EZ, CH, and AB: wrote the manuscript. EZ: conducted the experiments. EZ and ST: conducted the statistical analysis. AB and CH: supervision and grant support. All authors edited and reviewed the manuscript, as well as approved the submitted version.

## Conflict of Interest

The authors declare that the research was conducted in the absence of any commercial or financial relationships that could be construed as a potential conflict of interest.

## Publisher’s Note

All claims expressed in this article are solely those of the authors and do not necessarily represent those of their affiliated organizations, or those of the publisher, the editors and the reviewers. Any product that may be evaluated in this article, or claim that may be made by its manufacturer, is not guaranteed or endorsed by the publisher.
