# Supplementary figures and images for: Automated Home-Cage Monitoring During Acute Experimental Colitis in Mice
Source: Front Neurosci. 2021 Oct 22;15:760606. doi: 10.3389/fnins.2021.760606 (PMC8570043; doi:10.3389/fnins.2021.760606)

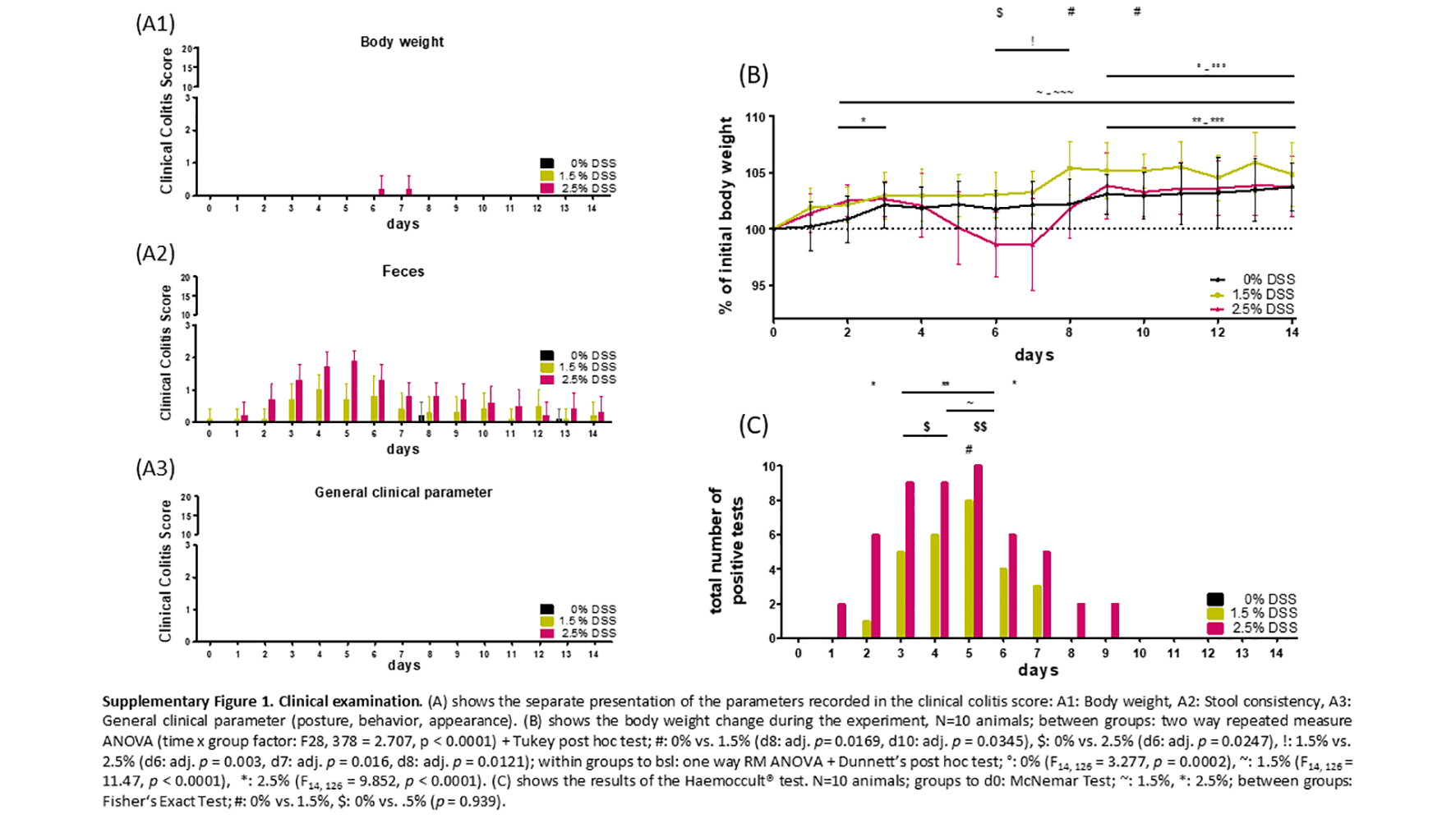

Supplement: Supplementary file 1 [file Image_1.TIF]

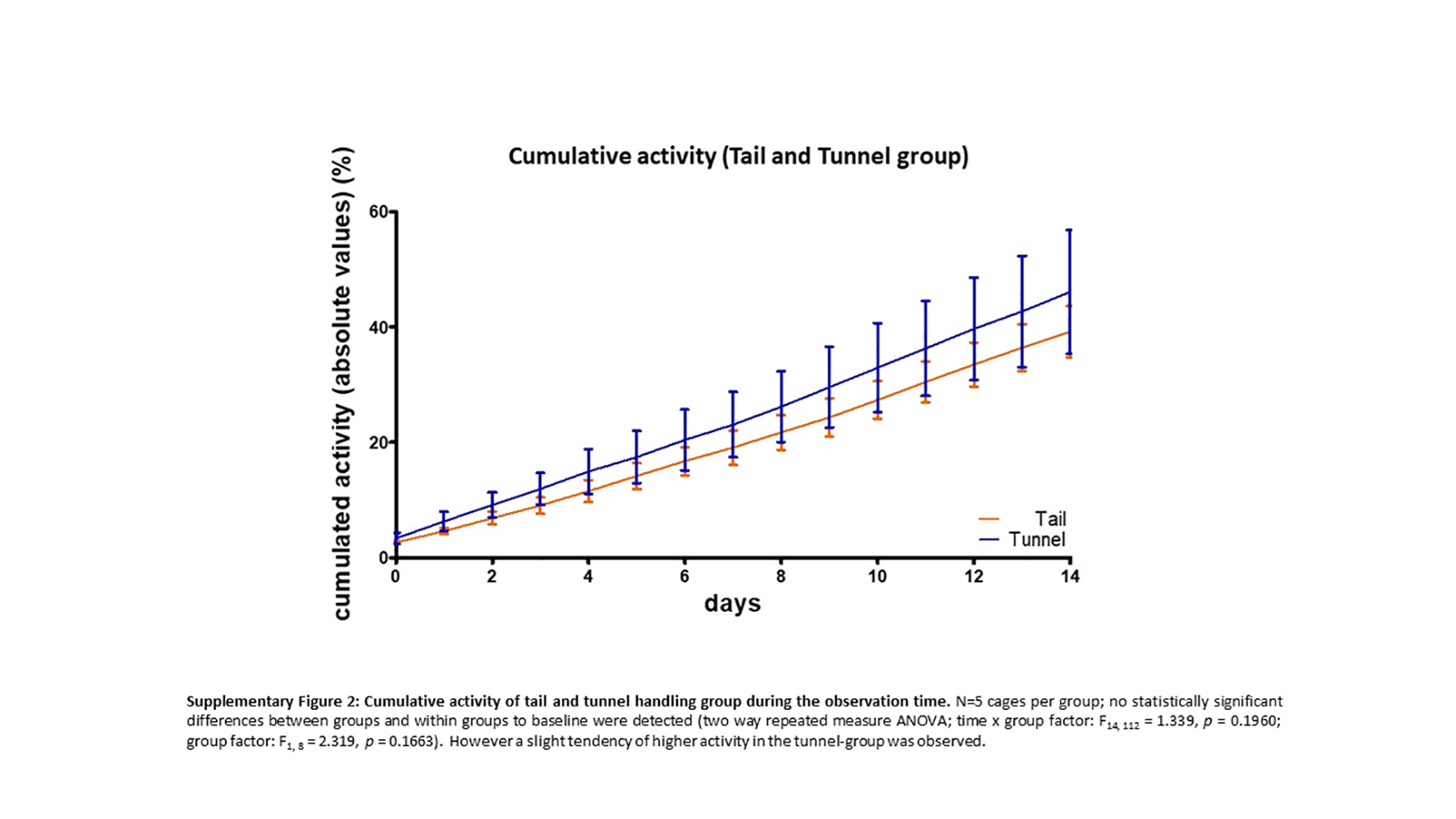

Supplement: Supplementary file 2 [file Image_2.TIF]

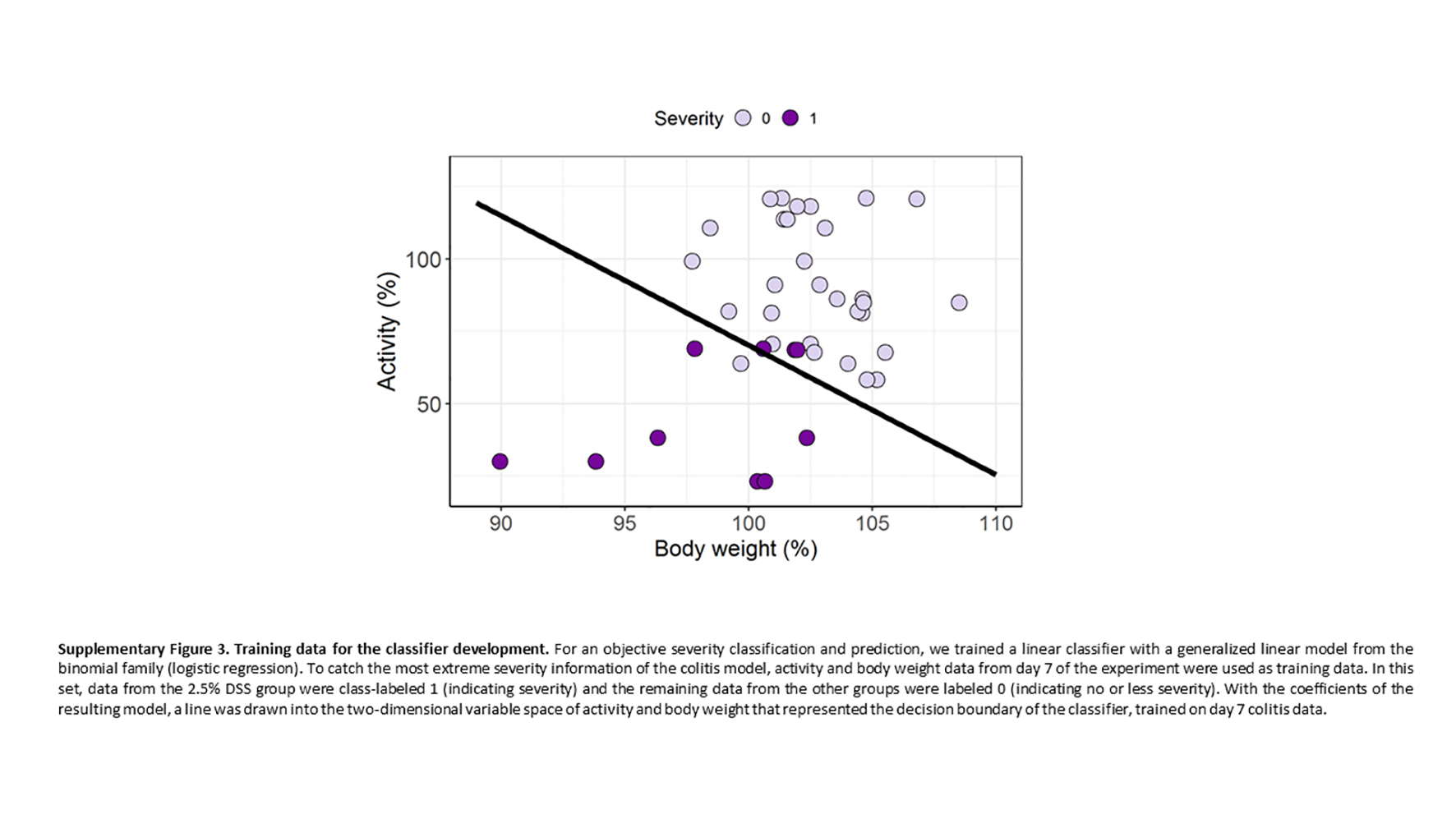

Supplement: Supplementary file 3 [file Image_3.TIF]

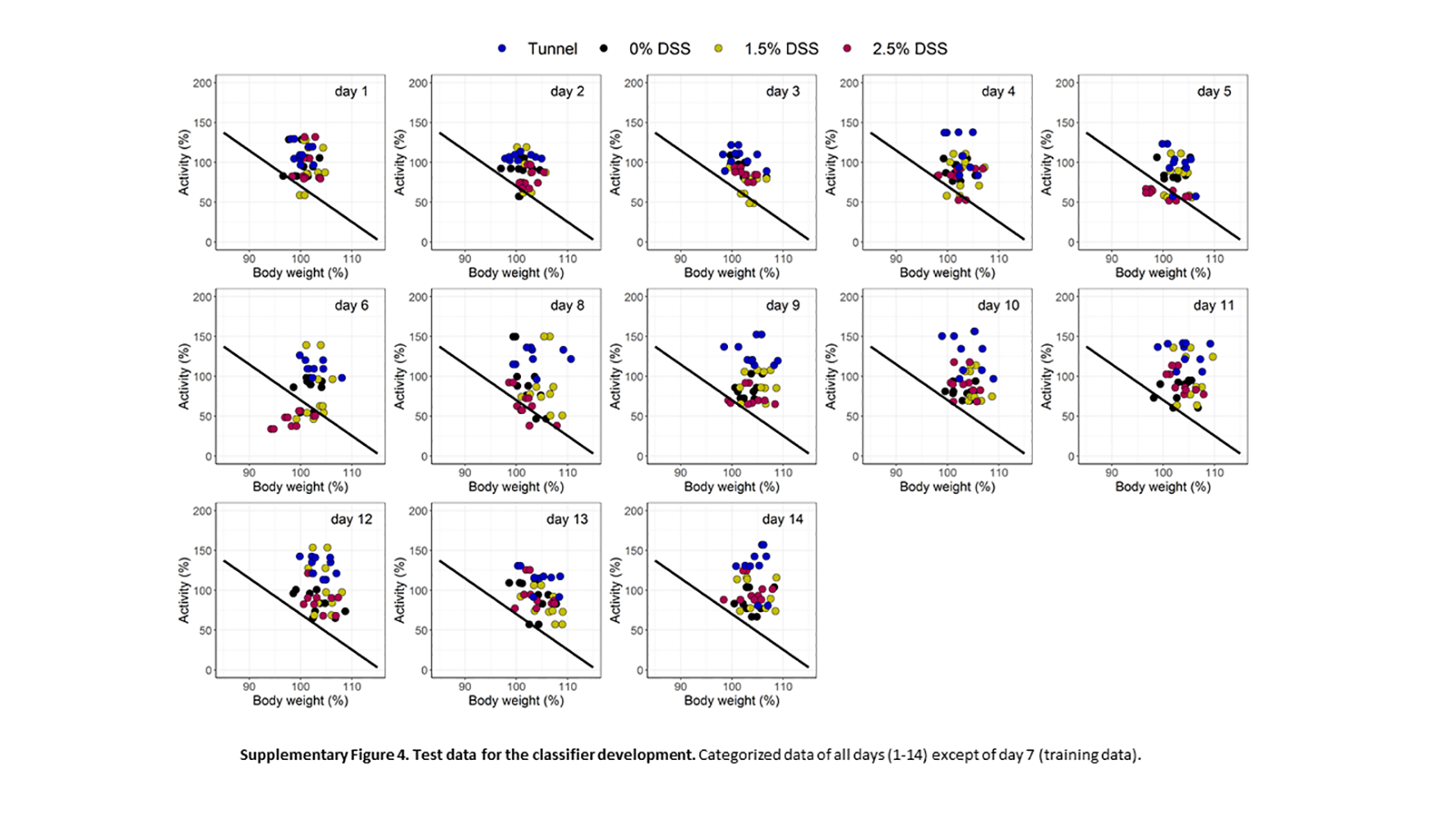

Supplement: Supplementary file 4 [file Image_4.TIF]
