# Supplementary material for: Automated Home-Cage Monitoring During Acute Experimental Colitis in Mice
Source: Front Neurosci. 2021 Oct 22;15:760606. doi: 10.3389/fnins.2021.760606 (PMC8570043; doi:10.3389/fnins.2021.760606)
Supplement: Supplementary file 5 [file Table_1.docx]

Supplementary Material

# Supplementary Tables

**Supplementary Table 1: Results of Nest Scoring**. The table shows the scoring results from day -13 until day 14 for all three DSS groups.
